# Multidisciplinary correlates of table tennis participation in children: a concept mapping study

**DOI:** 10.3389/fpubh.2025.1644306

**Published:** 2025-10-01

**Authors:** Jiang Liu

**Affiliations:** Jiangsu Food & Pharmaceutical Science College, Huai'an, China

**Keywords:** table tennis, motor performance, executive function, cognitive flexibility, child development, psychological resilience

## Abstract

**Background:**

Table tennis engages a combination of motor, cognitive, and psychosocial skills, demanding quick decision-making, precise coordination, and interpersonal interaction. This study explored the relationship between participation in table tennis training and improvements in children’s motor abilities, cognitive development, and psychological resilience.

**Methods:**

A total of 312 children (156 boys, 156 girls), aged 8–14 years, participated in a 12-month structured table tennis program. Motor performance was evaluated via agility drills, simple reaction time, and hand–eye coordination tasks. Cognitive outcomes were assessed using the Stroop Test and the Wisconsin Card Sorting Test (WCST). Psychosocial indicators encompassed self-efficacy, peer interactions, and perceived stress. Structural equation modeling (SEM) was employed to examine hypothesized direct and indirect associations among these domains.

**Results:**

Longer training duration was significantly associated with improved reaction time (*r* = −0.42, *p* < 0.001), agility (*r* = −0.38, *p* < 0.001), and hand-eye coordination (*r* = 0.46, *p* < 0.001). WCST errors (*r* = −0.38, *p* < 0.001) and Stroop response time (*r* = −0.42, *p* < 0.001) decreased. Self-efficacy (*r* = 0.41, *p* < 0.001) and social competence (*r* = 0.42, *p* < 0.001) increased, while perceived stress (*r* = −0.39, *p* < 0.001) and antisocial behavior (*r* = −0.43, *p* < 0.001) declined. Structural equation modeling revealed significant associations among motor, cognitive, and psychosocial outcomes.

**Conclusion:**

The findings indicate that structured table tennis training is associated with enhancements in motor coordination, executive functioning, and psychosocial well-being. These results highlight its potential value as an effective component of school-based developmental programs.

## 1 Introduction

Table tennis is a complex, high-speed sport that draws on fine motor control, rapid decision-making, and socially interactive play, making it a useful model for investigating multiple dimensions of child development. Although physical activity is broadly linked to physical and psychological benefits (1–3), the simultaneous engagement of motor, cognitive, and psychosocial systems in table tennis provides a distinctive context for examining their interplay. Preliminary evidence indicates improvements in motor coordination, reaction time, and balance among youth participants (3, 4); however, many studies evaluate these outcomes in isolation and often rely on self-report or narrowly defined experimental tasks, constraining a holistic understanding of developmental impact. Additional observational findings point to potential cognitive and affective benefits such as faster processing speed and lower cortisol levels (5) as well as better classroom focus and higher engagement in school-based physical education (6).

Owing to its dynamic demands simultaneous perceptual processing, rapid cognitive appraisal, and social–emotional regulation table tennis provides an ideal setting for examining interdependencies across developmental domains. Yet, unlike better-studied racquet sports such as tennis and badminton (7, 8), integrative analyses of table tennis remain limited (9–11), and no comprehensive framework delineates how behavioral domains interact during participation. To address this gap, the present study employs a concept-mapping approach to identify and visualize the multidimensional behavioral correlates of table tennis in school-aged children. Although prior work suggests that gameplay engages executive functions attentional control, inhibition, and working memory (12) and that contextual factors such as peer dynamics and socioeconomic conditions shape participation and training continuity (13, 14), systematic, child-focused investigations integrating motor, cognitive, and social behavior are scarce (2). Accordingly, we propose and test a holistic behavioral framework for table tennis participation.

Concept mapping provides a robust means to identify and visualize interrelated behavioral domains in this setting (6). In contrast to traditional regression or mediation techniques, it blends qualitative stakeholder input with quantitative clustering to uncover multidimensional structures spanning motor coordination, executive function, and social–emotional regulation. This approach is especially pertinent in child development research, where outcomes intersect and shift across developmental stages. Evidence from mixed-method education and health interventions shows that concept mapping can clarify how training activities translate into perceived and measurable developmental gains, thereby informing targeted curriculum design (6, 48).

Emerging evidence from tennis research underscores the importance of executive functions (EFs) in cognitively demanding racquet sports (7, 8). In an 18-month longitudinal study, Ishihara et al. (7) reported that higher EF indexed by cognitive flexibility predicted subsequent improvements in junior players’ competitive rankings, controlling for age and baseline performance. Extending these findings, Ishihara et al. (8) showed that greater tennis experience among children aged 6–12 was associated with stronger EF (inhibition, working memory, flexibility), which in turn predicted better dietary self-regulation; their SEM indicated an indirect path from tennis experience to healthy food intake via executive control. Collectively, these results highlight the broad developmental significance of EFs for sport-related performance and self-regulatory behaviors, reinforcing their relevance for sports such as table tennis.

Evidence from broader athletic literature reinforces the association between sport participation and executive function (EF), particularly in open-skill environments. Ren et al. (15) conducted a meta-analysis of 41 studies and found that athletes outperformed non-athletes in inhibitory control and working memory, with open-skill athletes (e.g., basketball, soccer) showing higher cognitive flexibility than those in closed-skill disciplines. Enriquez-Geppert et al. (16) similarly identified EF components, particularly working memory updating and set-shifting, as central to cognitive performance in multitasking contexts, which may be analogous to demands in fast-paced sports. While Barcelos et al. (17) emphasized EF impairments among combat athletes following concussions, their findings highlight the sensitivity of EF to intensive physical stress. Adithyan et al. (18) noted that elite teams have begun using AI-based systems to monitor and train attention, task switching, and decision-making speed. Although Martinez (19) did not assess EF directly, the study noted demographic patterns in skill-based sports that suggest a sociocognitive dimension to performance. Mendes et al. (49) further proposed that IGF1 gene polymorphisms linked to neuroplasticity may play a role in physical and cognitive adaptability, though without direct EF measurement.

The current status of research suggests that cognitively demanding, open-skill sports like table tennis may be meaningfully associated with executive function and other developmental indicators in youth. However, most prior work has examined these domains in isolation, limiting our understanding of how motor, cognitive, and psychosocial processes interrelate. To address this gap, the present study investigates behavioral correlates of table tennis participation in school-aged children using a concept mapping framework. This approach enables the integration of multiple behavioral domains and allows for the visualization of complex interdependencies without assuming direct causality.

### 1.1 Table tennis participation among children

Table tennis has been shown to enhance agility, coordination, and motor skills, although existing studies focus predominantly on elite populations, limiting their relevance to general youth cohorts (20–23). Zhao et al. (3) reported reaction time improvements of 15.3% and coordination gains of 9.7% in elite players but failed to address social or cognitive outcomes. Gu et al. (24) demonstrated motor benefits in preschoolers (+8.4% reaction speed, +6.2% hand-eye coordination), yet the lack of longitudinal follow-up raised concerns about sustained effects.

Table tennis may also support visual development. Liu et al. (11) identified it as among the most effective sports for mitigating myopia progression (SUCRA = 84.1). However, their study lacked controls for confounding variables like screen time and outdoor activity. Luca et al. (25) found enhanced cardiovascular efficiency (+8.9%) and oxygen uptake (+6.4%) in children with congenital heart disease, suggesting table tennis is suitable for pediatric rehabilitation. However, its comparative advantages over other racket sports remain unclear. While these studies support the physical merits of table tennis, most rely on structured interventions, overlooking informal participation and the longitudinal trajectory of skill development.

### 1.2 Motor skills, cognitive development, and social engagement

Despite engaging multiple domains, executive function, emotion regulation, and social interaction, table tennis research has rarely integrated these factors systematically (11, 26–28) documented gains in cognitive flexibility (+10.7%) and executive function (+9.2%) in overweight adolescents, yet did not explore motor-cognitive interdependence. Although their cross-sectional design limits causal interpretation, Martin-Rodriguez et al. (5) observed a 22.4% cortisol reduction, indicating stress regulation benefits. Gonzalez-Devesa et al. (12) reported peer interaction improvements (+17.8%) in children with ASD, though heterogeneous intervention protocols hinder generalization. Similarly, Rocliffe et al. (6) found that structured school-based table tennis increased physical activity engagement (+26%), but did not measure outcomes beyond institutional settings.

Early childhood studies have highlighted promising outcomes but suffer methodological limitations. Gu et al. (24) showed gross motor improvements (+12.5% object control, +10.2% locomotion, +9.3% balance), yet lacked post-intervention tracking. Salmela (29) reported a 14% gain in short-term recall through movement-based learning, though no objective metrics validated these findings. The now-retracted study by Qijie et al. (30) previously claimed increases in self-esteem (+21.3%) and self-efficacy (+18.6%) among left-behind children, but serious methodological flaws undermined its credibility. These issues underscore the need for rigorous, integrative research on psychosocial dimensions. Our study responds by using concept mapping to map these domains systematically and identify robust interdependencies.

### 1.3 Concept mapping in table tennis

Concept mapping enables structured visualization of complex behavioral relationships, yet its application in sports science remains limited. Rocliffe et al. (6) used it to analyze school-based sports, demonstrating improved engagement (+18%) and motor skills (+11%), but neglected cognitive and social dimensions. Wang et al. (31) applied fNIRS and found neuroplasticity gains (+34.2% postural stability) in rehabilitation contexts, although their study lacked scalability for general populations.

Prior studies often remain siloed within specific domains. Khudair et al. (32) examined self-regulation, while Ochs et al. (33) explored motor classification without integrating findings into a unified framework. Neto et al. (34) demonstrated that Wii-based table tennis improved reaction time (+10.6%), but with limited evidence of transfer to real-world motor performance. Driban et al. (35) concluded no increased osteoarthritis risk in racket sports, though biomechanical differences across sports were not considered. These fragmented findings reveal a critical gap in integrated modeling.

Our study addresses this need by applying concept mapping within a systems theory and multidimensional learning framework to examine how motor, cognitive, and social variables interact in table tennis. This approach provides a structured, data-driven model that enhances understanding developmental outcomes and informs training design and educational policy.

### 1.4 Hypothesis

Table tennis participation is expected to be associated with measurable interrelations among motor skills, executive function, and social engagement in children, which can be systematically captured using a concept mapping framework.

### 1.5 Objectives

1. Identify key physical activity behaviors associated with table tennis participation.
2. Analyze the correlations between motor skills, cognitive function, and social engagement.
3. Utilize concept mapping to represent these interconnections and optimize training programs visually.

## 2 Methods

### 2.1 Study design and setting

This cross-sectional, mixed-methods study was conducted from January to July 2024 across three schools in Nanjing, China. Quantitative motor, cognitive, and social skill assessments were integrated with qualitative concept mapping. All procedures adhered to STROBE guidelines. Data collection occurred in controlled gym and lab settings (22–24 °C, 40–50% humidity), with cognitive tests administered in distraction-minimized environments. Each child completed two 90-min sessions, spaced 48 h apart. All cognitive and motor tests followed a fixed sequence across participants, administered at identical time windows (9:00–11:30 AM) to control for circadian variation. Environmental conditions, testing scripts, and task order were standardized by trained assessors using a uniform protocol to ensure inter-session consistency.

### 2.2 Participants and eligibility criteria

Participants were recruited via school-based flyers, classroom presentations, and parental outreach (see Table 1). Researchers hosted in-person briefings, and enrollment was confirmed within a two-week window. Stratified sampling ensured equal representation of boys and girls. Sample size was determined using G*Power (*f* = 0.25, power = 0.90, *α* = 0.05), yielding a minimum of 310; the final sample comprised 312 children (156 boys, 156 girls), aged 8–14 years.

**Table 1:** Inclusion and exclusion criteria.

Inclusion criteria	Exclusion criteria
Aged 8–14 years	Musculoskeletal injuries in the past 6 months
Enrolled in schools with table tennis programs	Diagnosed with ADHD or ASD
Training ≥3 times per week for ≥6 months under certified coaches	Competitive training in other racket sports
No neurological, orthopedic, or cognitive impairments (verified via PPHQ adapted from AAP PPE)	Uncorrectable vision worse than 20/40 on Snellen Test
Ability to complete assessments independently and pass visual processing pre-screening	Color vision deficiency (failed Ishihara Test)
Provided written parental consent and child assent	Missed ≥25% of scheduled sessions

### 2.3 Ethics statement

Ethical approval was obtained from the Nanjing University Ethics Committee (Protocol No. 2024-TT-0412). Written informed consent was obtained from parents/guardians, and verbal assent was secured from all child participants before enrollment.

### 2.4 Brainstorming

Participants, educators, and coaches joined 60-min brainstorming sessions (6–10 people/group), facilitated by trained moderators. Discussions focused on motor, cognitive, and social influences on table tennis, yielding consolidated thematic lists via qualitative thematic coding. Using Minds21 Software (Severens, Netherlands), participants individually grouped correlates, labeled clusters, and explained classifications. A similarity matrix and hierarchical cluster analysis (*κ* = 0.89, *p* < 0.001) ensured high inter-rater reliability. Cluster labels were evaluated via 5-point Likert ratings and revised through consensus when ≥70% of participants disagreed. Final label agreement reached 92.3%. Children participated in a 20-min guided familiarization session using concrete examples and cartoon analogies to ensure age-appropriate engagement in clustering and labeling. Moderators used visual aids, think-aloud modeling, and simplified language to scaffold understanding of similarity judgments and thematic grouping. Labeling tasks were co-reviewed by adults and children to confirm mutual comprehension, ensuring cognitive readiness without overburdening abstraction capacities.

#### 2.4.1 Rating, scaling, and cluster formation

Each correlate was rated on two 5-point Likert scales assessing modifiability (1 = Not Modifiable, 5 = Highly Modifiable) and effect size (1 = No Effect, 5 = Very High Effect). A familiarization session ensured consistency in interpretation. Multidimensional Scaling (MDS) visualized conceptual distances in two-dimensional space, followed by Ward’s HCA using Euclidean distances to define clusters. Scree plot inflection points and silhouette coefficients (>0.70) confirmed cluster consistency.

### 2.5 Motor skill assessment

Participants wore standardized sports attire and table tennis footwear. A 10-min dynamic stretching routine and 5 min of table tennis-specific drills (lateral footwork, ball-tracking) preceded testing (see Supplementary Figures S4, S5).

#### 2.5.1 Reaction time test

Reaction speed was assessed using a computerized reaction-time device (Lafayette Instruments, USA). Participants sat with their dominant hand on a response button (10 cm midline) and focused on a high-resolution screen. They completed 20 trials responding to randomly timed visual and auditory stimuli, with 3.0 s inter-trial intervals randomized. Mean response latency (ms) was recorded, excluding trials exceeding two standard deviations from the mean.

#### 2.5.2 Agility test

The Illinois Agility Test was conducted using electronic timing gates (Brower Timing Systems, USA) on a 10 m × 5 m course. Four cones were placed at 3.3 m intervals, with four additional directional cones enforcing lateral movement. Three timed trials were recorded, with the fastest time (s) used for analysis. A 90-s passive recovery period was provided between attempts.

#### 2.5.3 Hand-eye coordination test

A Gazelle Sports ball-tracking system assessed visual-motor synchronization and reaction accuracy. Participants stood 2 m from a screen displaying random target zones and struck a ball with a motion-sensor-equipped paddle (Gazelle MX-200, China). A high-speed camera (Sony RX10 IV, 960 fps) recorded contact points, response latency, hit accuracy (%), and movement variability (CV%).

#### 2.5.4 Balance test

Postural stability was assessed using an AMTI force platform (USA, 1000 Hz), recording center-of-pressure (CoP) displacement under three 30-s conditions: eyes open, eyes closed (both with feet together), and single-leg stance with the opposite foot elevated 10 cm. CoP displacement was measured in anteroposterior and mediolateral directions (mm), with triplicate measurements per condition.

### 2.6 Cognitive assessments

#### 2.6.1 Wisconsin card sorting test (WCST)

**Figure 1:**
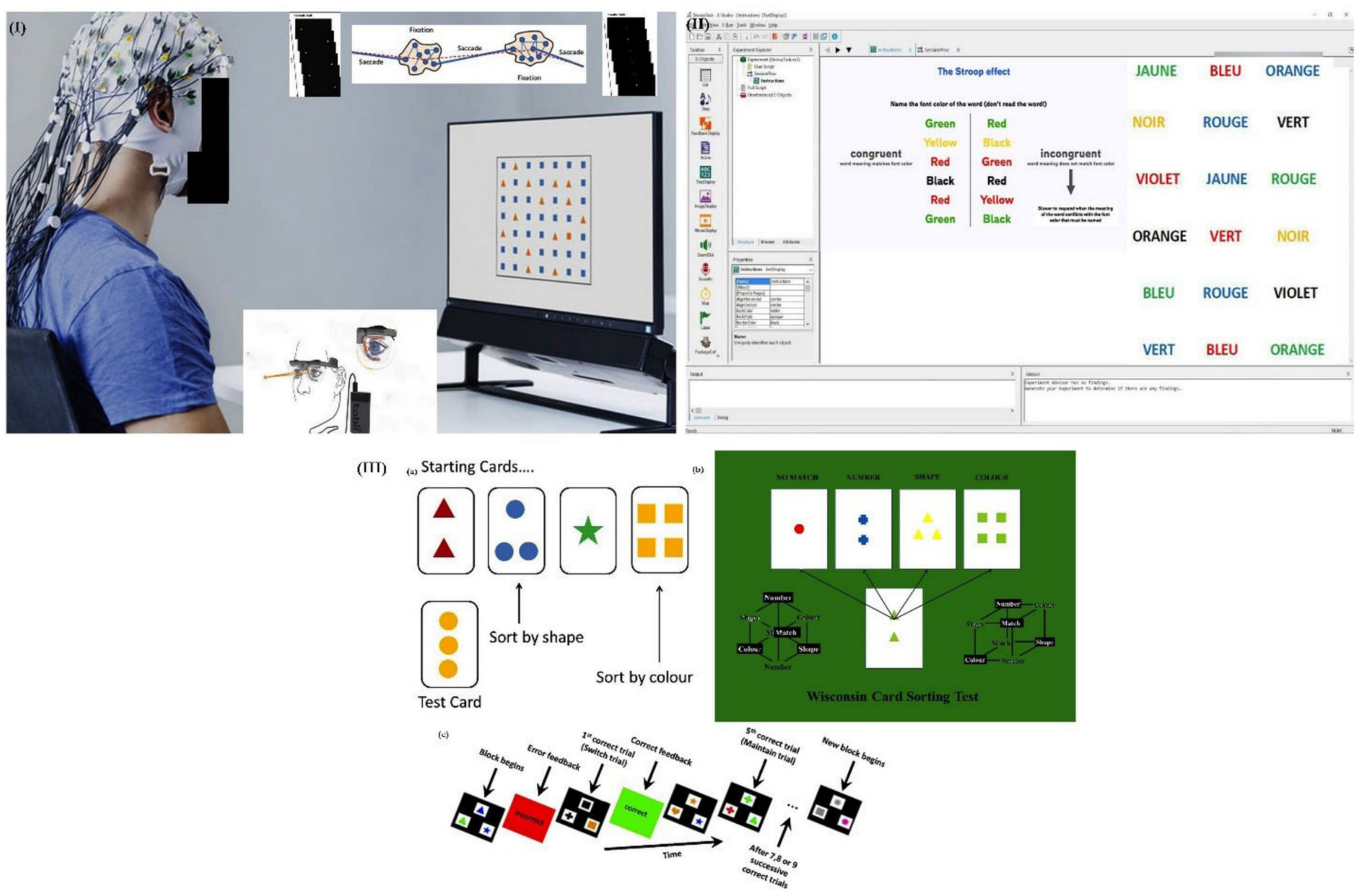
Experimental setup for cognitive assessments, including (I) eye-tracking system for visual processing, (II) Stroop test interface for response inhibition, and (III) WCST for cognitive flexibility and rule-switching.

Cognitive flexibility and problem-solving were assessed using a computer-based WCST (PsyToolkit, Netherlands) (see Figure 1). Participants viewed four key cards differing in color, shape, and number, and sorted stimulus cards based on an unstated rule that shifted unpredictably. The test required continuous adaptation, with automated feedback provided. Metrics included total errors, perseverative errors, and completion time (s). The WCST was administered using a version normed on young Chinese children (36).

#### 2.6.2 Stroop test

The digital Stroop Task (E-Prime 3.0, Psychology Software Tools, USA) was administered in a dimly lit, soundproofed room to minimize distractions (see Figure 1). A calibrated 24-inch monitor (BenQ, Taiwan) displayed color-word stimuli (e.g., “RED,” “BLUE,” “GREEN”) in incongruent ink colors, requiring participants to name the ink color while suppressing the urge to read the word. Three 2-min trials were completed. Response times (ms), error rates (%), and Stroop interference scores (difference between congruent and incongruent conditions) were recorded. The Stroop task employed the Mandarin Victoria version, previously shown to retain equivalent psychometric properties including test–retest reliability relative to the original version in Chinese-speaking population (37).

**Figure 3:**
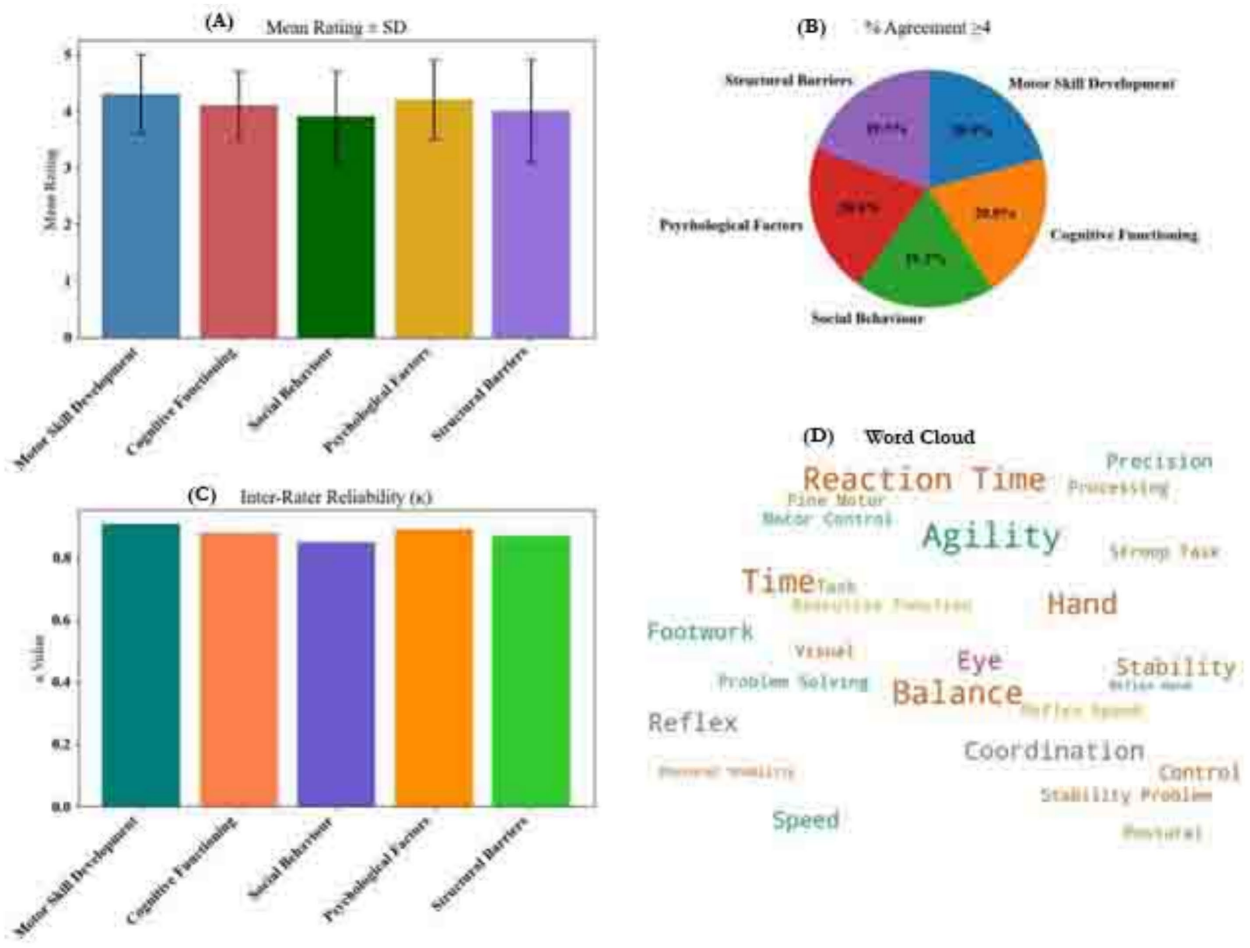
Brainstorming analysis **(A)** bar chart of mean ratings ± SD for each cluster, **(B)** pie chart of percentage agreement ≥ four across clusters, **(C)** bar chart of inter-rater reliability (κ) per cluster, and **(D)** word cloud of key correlates associated with the thematic clusters.

#### 2.6.3 Eye-tracking analysis

Visual processing was assessed using the Tobii Pro Spectrum system (Tobii Technology, Sweden), which recorded fixation stability, saccadic latency, and anticipatory gaze. Participants tracked circular targets appearing randomly on a gray background, following predictable (smooth pursuit) and unpredictable (saccadic) paths. Metrics included gaze duration (ms), pursuit accuracy (%), and stimulus latency (ms). Eye-tracking methods were modeled on studies that characterized gaze behavior in Chinese children during reading (31, 38).

### 2.7 Psychometric measures

The Self-Efficacy Scale for Children (SESC), measuring academic and social self-efficacy, has demonstrated acceptable construct validity and internal consistency (Cronbach’s *α* ≈ 0.80–0.83) in early school-aged children (50). The Perceived Stress Questionnaire (PSQ), validated in both English and Italian-speaking populations, shows high reliability (α > 0.90) and strong test–retest stability (*r* ≈ 0.82) (51). In our study, Task instructions and items were translated and pilot-tested (*n* ≈ 30) to ensure clarity and relevance in the local context.

The School Social Behavior Scales-Second Edition (SSBS-2) assesses social competence and antisocial behavior, with Cronbach’s alpha ranging from 0.96 to 0.98 and high inter-rater reliability in school-aged children (39, 52, 53). Video-coded behavioral tracking (using Dartfish 360) captured interactions such as eye contact, verbal turn-taking, and cooperative play through an adapted ethogram.

### 2.8 Concept mapping

Concept mapping was selected over traditional exploratory factor analysis (EFA) or principal component analysis (PCA) due to its suitability for integrating both qualitative themes and quantitative ratings in child populations. Concept mapping accommodates subjective input, unlike EFA or PCA, which assume linear relationships and require large-scale variable sets. It provides intuitive visualizations of perceived interrelations, aligning with children’s developmental level and the interdisciplinary nature of this study. Final concept maps were developed using a three-stage process integrating Hierarchical Clustering Analysis (HCA), Multidimensional Scaling (MDS), and Structural Equation Modeling (SEM) with Minds21 Concept Mapping Software (Severens, Netherlands) and SPSS AMOS (IBM, USA). SEM was applied to quantify associations among behavioral constructs within the cross-sectional dataset. All directional paths were specified based on theoretical alignment rather than temporal ordering, and results were interpreted as correlational patterns without causal attribution.

HCA identified thematic clusters based on relational strength using Ward’s method and Euclidean distances, validated through silhouette coefficients (>0.70). MDS positioned correlates spatially in a two-dimensional solution (stress <0.15), with axes representing influence and modifiability. SEM quantified predictive pathways between motor, cognitive, and social variables, testing direct and indirect effects using MLE, with model fit assessed via CFI (>0.90), TLI (>0.90), and RMSEA (<0.08). To ensure conceptual clarity, three distinct constructs were defined and analyzed in alignment with the structural equation modeling framework. Psychological resilience was defined as the capacity to adaptively regulate affective responses and maintain functional coping under perceived stress, measured using the Psychological Stress Questionnaire (PSQ-20). Psychosocial development refers to observable interpersonal behaviors, including cooperation, peer engagement, and reduction of antisocial conduct, and it is assessed via the School Social Behavior Scales–Second Edition (SSBS-2). In contrast, psychological growth denoted cumulative internal changes in emotional regulation and self-reflection over time, operationalized through latent modeling of self-efficacy gains, affective stability, and behavioral persistence. Each construct was mapped to distinct domains within the SEM framework to preserve theoretical independence and avoid construct conflation.

### 2.9 Statistical analysis

Data analysis was conducted using SPSS 28.0 (IBM, USA), R 4.2.2 (R Foundation, Austria), and NVivo 14 (QSR International, Australia). Assumption checks included normality (Shapiro–Wilk), homoscedasticity (Levene’s test), and multicollinearity (VIF < 5). Descriptive statistics (M, SD, 95% CI) were computed. Independent t-tests compared motor, cognitive, and social measures across groups. Multiple regressions adjusted for age, training history, SES, BMI, and visual acuity, predicting reaction time, agility, Stroop interference, and self-efficacy. Standardized *β* and adjusted R^2^ were reported, with significance at *p* < 0.05 (two-tailed). Structural Equation Modeling (SEM) in SPSS AMOS (IBM, USA) tested direct and indirect effects across latent variables using maximum likelihood estimation. Model fit was evaluated using χ^2^/df (<3.0), CFI (>0.90), TLI (>0.90), and RMSEA (<0.08). Missing data (<3%) were handled using full information maximum likelihood (FIML) in SEM and pairwise deletion for descriptive and regression analyses. Data patterns were confirmed to be missing at random (Little’s MCAR test, *p* > 0.10).

## 3 Results

**Figure 2:**
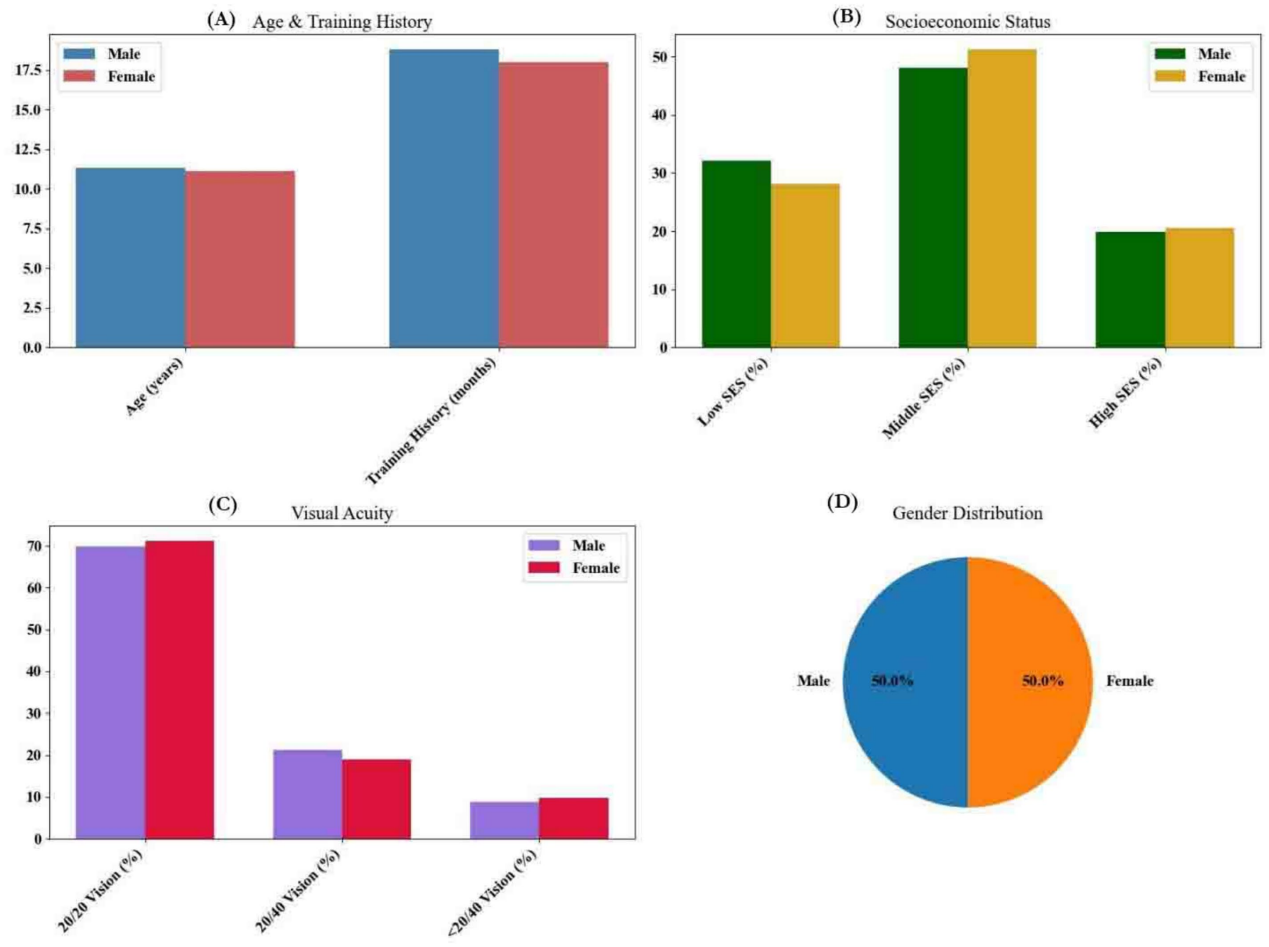
**(A)** Distribution of participant ages and training duration by gender, **(B)** percentage of participants in low, middle, and high socioeconomic status categories, **(C)** distribution of participants based on Snellen vision scores, and **(D)** proportion of male and female participants using a pie chart.

The mean age of participants was 11.2 ± 2.1 years, with boys and girls exhibiting similar distributions (see Figure 2). Training history averaged 18.4 ± 7.6 months, with boys training slightly longer than girls (18.8 ± 7.4 vs. 18.0 ± 7.8 months). Socioeconomic status distribution showed 32.1% of boys and 28.2% of girls in the low-income category, while 48.1% of boys and 51.3% of girls were from middle-income backgrounds. Visual acuity assessments indicated ~70% had 20/20 vision, while ~9% had vision worse than 20/40. All *p*-values were adjusted using the Benjamini-Hochberg false discovery rate (FDR) method for multiple comparisons. Findings reported as statistically significant survived FDR-corrected thresholds of q < 0.05 unless otherwise noted.

Males and females exhibited broadly comparable performance across motor, cognitive, and social domains. However, females demonstrated slightly greater Stroop interference reduction (*Δ* = 23.2 ms, *p* = 0.042) and higher cooperation scores on SSBS-2 (*p* = 0.038), while males outperformed on agility tests (Δ = 0.7 s, *p* = 0.049). No significant interactions between sex and training duration were observed in regression or SEM models, and subgroup comparisons were exploratory to avoid confounding. Age-stratified analyses revealed distinct trends: children aged 11–14 years showed stronger training-related associations with executive functioning (e.g., WCST completion time, Stroop interference), whereas younger children (8–10 years) showed greater gains in motor coordination (e.g., agility, balance). These effects are summarized in Supplementary Table S1.

Participants identified five thematic clusters influencing table tennis participation. Motor Skill Development received the highest agreement (*M* = 4.3, *κ* = 0.91), reflecting strong consensus around the role of agility, coordination, and balance in youth engagement (Figure 3). Cognitive Functioning (*M* = 4.1, *κ* = 0.88), including executive processes like working memory and attention, was also rated highly. Psychological Factors (*M* = 4.2) and Social Behavior (*M* = 3.9) were perceived as essential for motivation and peer interaction, while Structural Barriers (*M* = 4.0) highlighted concerns around access, equipment, and cost. All clusters showed high inter-rater reliability (*κ* ≥ 0.85), indicating stable categorization.

Hierarchical clustering analysis (HCA) condensed these into three broader macro-clusters. Motor Performance achieved the strongest agreement (κ = 0.89) and the highest cluster stability (Supplementary Figure S1), underscoring its foundational role in participation. Cognitive Processing and Psychosocial Development followed closely in internal consistency. Motor-related clusters also had the highest rating consensus (87% ≥ 4), whereas cognitive and psychosocial clusters, while still rated highly, showed slightly more variability. Final cluster labels were retained following participant consensus review, with label agreement exceeding 90% across domains (Supplementary Figure S2).

Reaction time and agility test performance were negatively correlated with training duration (*p* < 0.001) (Table 2). Hand-eye coordination metrics varied: hit accuracy positively correlated (*r* = 0.46, *p* < 0.001), while response latency was negatively correlated (*r* = −0.41, *p* < 0.001). Movement variability showed a moderate positive correlation (*r* = 0.36, *p* < 0.001).

**Table 2:** Motor skill performance and correlations with training duration.

Test	Mean	*r*	Sig.	95% CI (r-values)
Reaction Time (ms)	321.5 ± 45.2	−0.42	<0.001***	[−0.55, −0.28]
Agility Test (s)	12.3 ± 1.8	−0.38	<0.001***	[−0.51, −0.24]
Hand-Eye Coordination				
Hit Accuracy (%)	85.6 ± 7.2	0.46	<0.001***	[0.32, 0.58]
Response Latency (ms)	255.4 ± 38.7	−0.41	<0.001***	[−0.54, −0.27]
Movement Variability (CV%)	12.8 ± 3.1	0.36	< 0.001**	[0.21, 0.50]

**p < 0.01, ***p < 0.001.

Postural stability was inversely correlated with training duration (Supplementary Table S2). CoP displacement decreased with training, both with eyes open (*r* = −0.39, *p* < 0.001) and closed (*r* = −0.42, *p* < 0.001). Single-leg stance showed similar trends on the dominant (*r* = −0.44, *p* < 0.001) and non-dominant leg (*r* = −0.46, *p* < 0.001). Mediolateral and anteroposterior CoP displacement also declined (*r* = −0.40, *r* = −0.43, *p* < 0.001), indicating improved stability with more prolonged training exposure.

**Figure 4:**
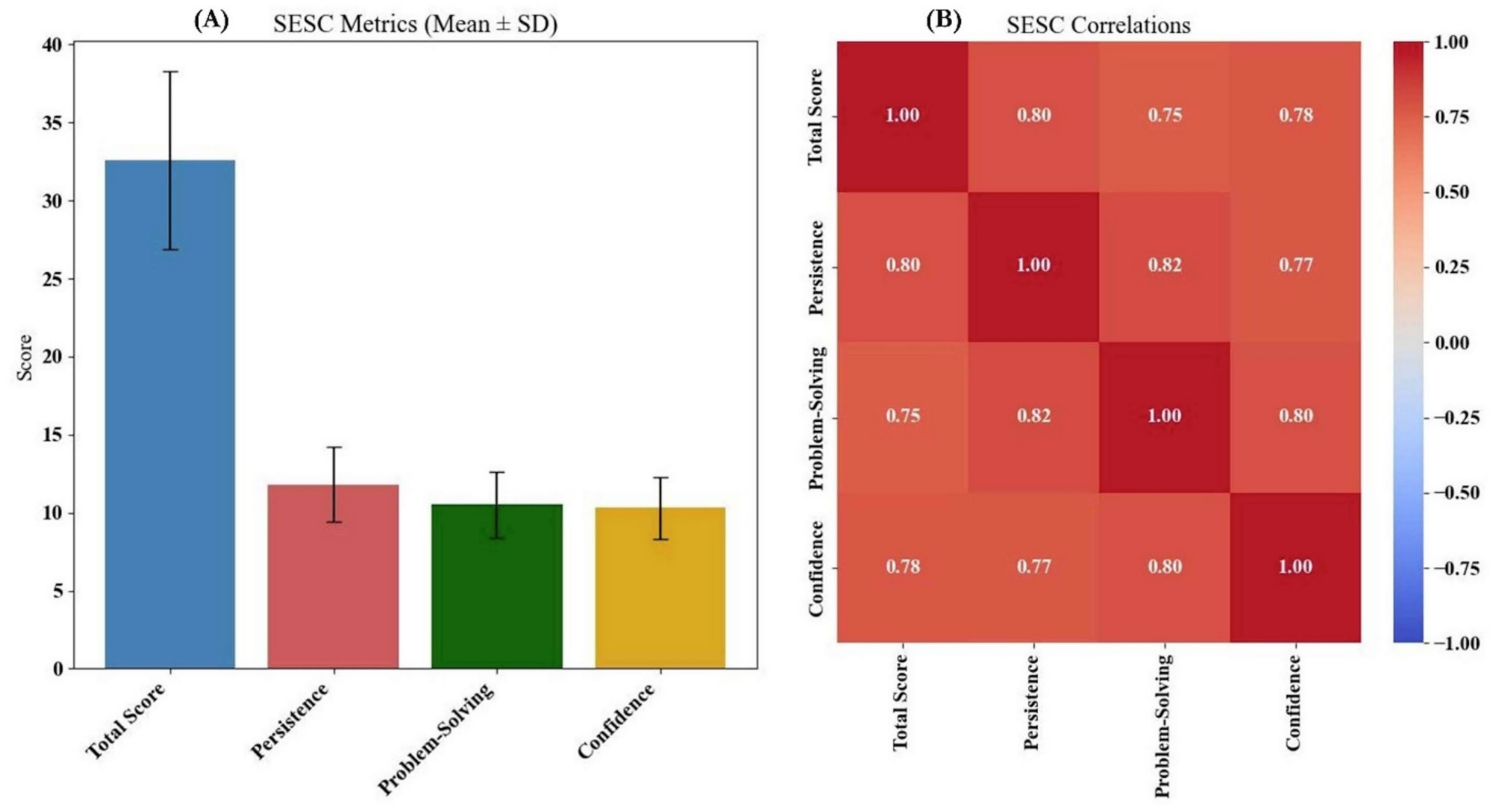
**(A)** Cognitive and psychological performance and WCST metrics, and **(B)** WCST correlation matrix.

**Figure 5:**
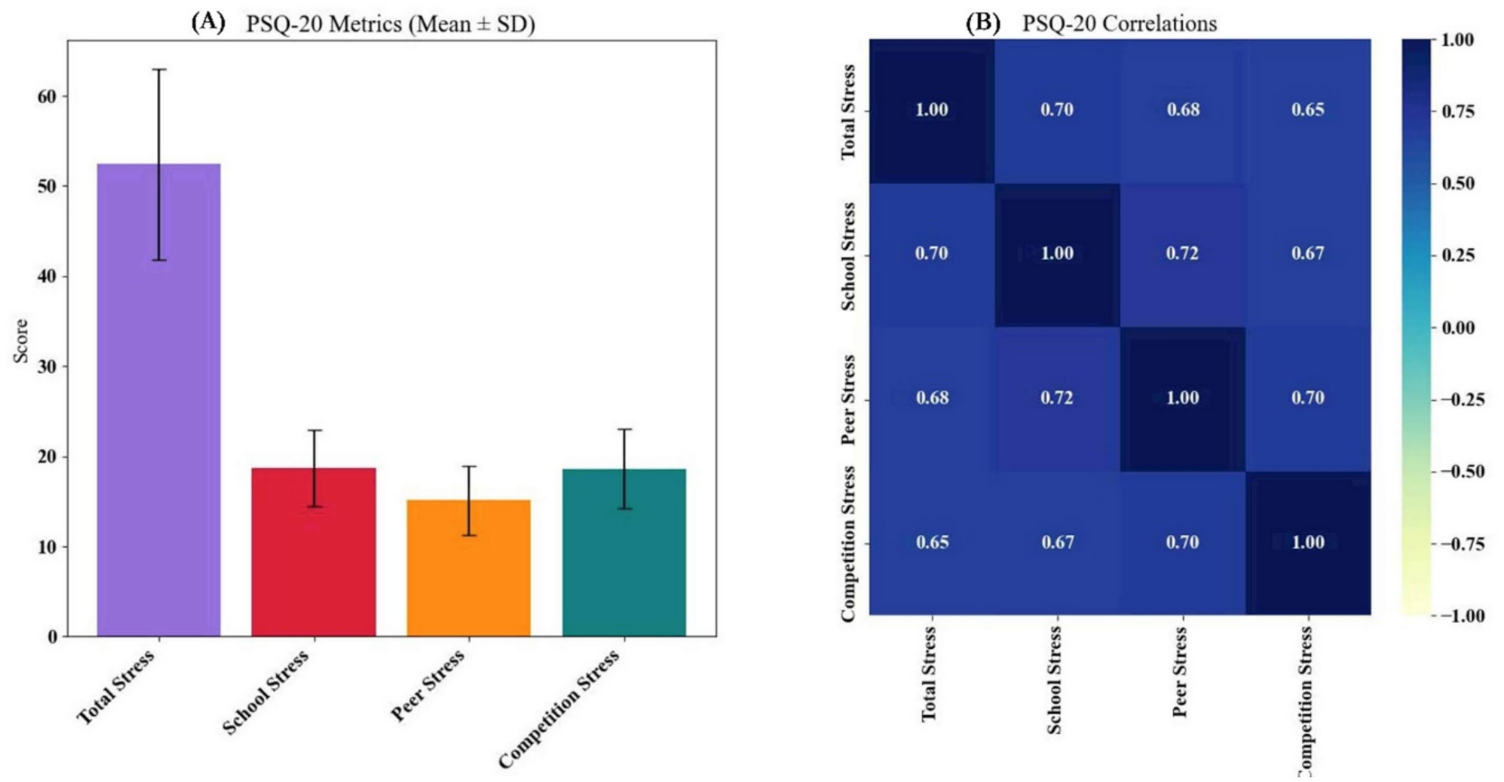
**(A)** Stroop test metrics and **(B)** Stroop test correlation matrix.

**Figure 6:**
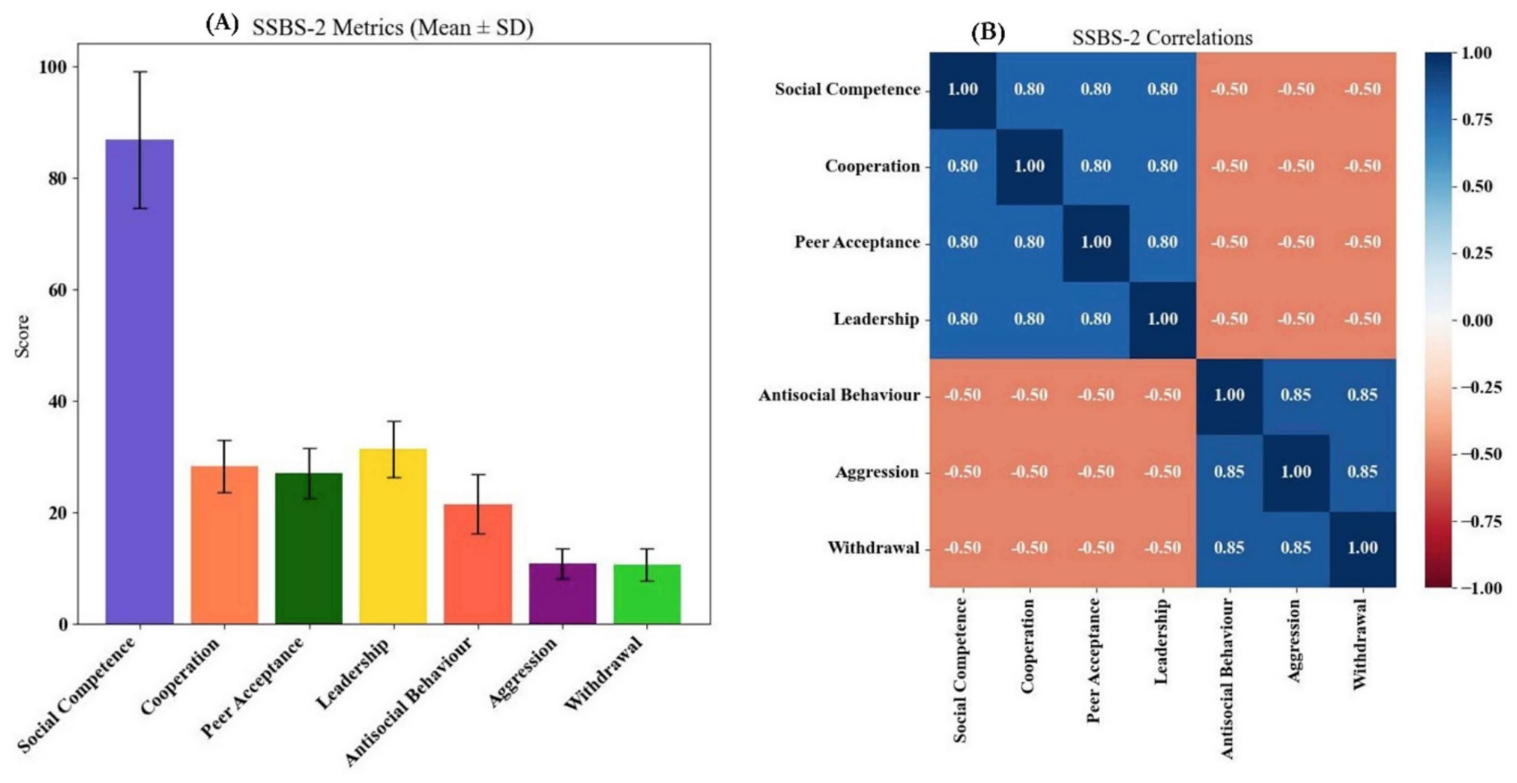
**(A)** Eye-tracking metrics and **(B)** Eye-tracking correlation matrix.

Cognitive and psychological performance showed consistent associations with training duration. On the WCST, reductions in total and perseverative errors, as well as faster completion times (r values ranging from −0.35 to −0.40, *p* < 0.001), suggested improved cognitive flexibility and rule-shifting ability (Figure 4). Similar patterns emerged on the Stroop task, where reduced response times and interference effects (*r* ≈ −0.45) indicated enhanced attentional control (Figure 5). Eye-tracking metrics further supported these findings: children with more training exhibited better anticipatory gaze and fixation stability (*r* > 0.39), along with faster saccadic shifts, reflecting more efficient visual processing (Figure 6).

Psychological self-efficacy (SESC) improved with training (*r* = 0.41), especially in persistence and problem-solving domains. Stress, as measured by the PSQ-20, decreased across academic, peer, and competition-related dimensions (*r* ≈ −0.39), suggesting better emotional regulation. Social competence (SSBS-2) was higher among more experienced participants (*r* = 0.42), with notable gains in cooperation and peer engagement. Conversely, antisocial behaviors including aggression and withdrawal declined as training increased (*r* ≈ −0.43), pointing to more adaptive social adjustment (Supplementary Figure S3).

Longer training duration was a strong predictor of improved psychosocial outcomes, including higher self-efficacy (*β* = 0.39) and social competence (*β* = 0.41), along with lower antisocial behavior (*β* = −0.38) (Table 3). Socioeconomic status and peer acceptance contributed uniquely to self-efficacy, while competition-related stress elevated perceived stress levels, countered by a protective effect of SES. Social competence was enhanced by cooperation and peer acceptance, whereas aggression and withdrawal significantly predicted greater antisocial tendencies. All effects were statistically significant (*p* < 0.001), highlighting a coherent pattern of behavioral adaptation with extended training exposure.

**Table 3:** Multiple regression analysis predicting psychological and social outcomes from training duration.

Dependent variable	Predictor	*β*	SE	t-value	*p*-value	95% CI
Self-Efficacy (SESC)	Training Duration (Months)	0.39	0.06	6.18	<0.001***	[0.27, 0.51]
	Socioeconomic Status	0.22	0.05	4.33	< 0.001**	[0.11, 0.34]
	Peer Acceptance	0.28	0.07	5.02	<0.001***	[0.17, 0.40]
Perceived Stress (PSQ-20)	Training Duration (Months)	−0.36	0.07	−5.69	<0.001***	[−0.48, −0.24]
	Competition Stress	0.34	0.06	5.28	<0.001***	[0.22, 0.46]
	Socioeconomic Status	−0.19	0.05	−3.82	< 0.001**	[−0.30, −0.08]
Social Competence (SSBS-2)	Training Duration (Months)	0.41	0.06	6.42	<0.001***	[0.29, 0.53]
	Cooperation	0.33	0.07	5.71	<0.001***	[0.21, 0.45]
	Peer Acceptance	0.30	0.06	5.27	<0.001***	[0.19, 0.42]
Antisocial Behavior (SSBS-2)	Training Duration (Months)	−0.38	0.07	−5.92	<0.001***	[−0.51, −0.25]
	Aggression	−0.33	0.06	−5.38	<0.001***	[−0.45, −0.21]
	Withdrawal	−0.31	0.07	−5.02	<0.001***	[−0.44, −0.19]

Regression coefficients (β) are standardized. Higher training duration was associated with higher self-efficacy and social competence, while negatively associated with stress and antisocial behavior. **p < 0.01. ***p < 0.001.

Structural equation modeling revealed a coherent pathway linking physical, cognitive, and psychological domains (Table 4). Motor performance strongly predicted cognitive function (*β* = 0.49), which in turn contributed to greater psychological resilience (*β* = 0.31). Social engagement provided an additional positive influence on resilience (*β* = 0.28) and was itself predicted by motor performance (*β* = 0.37). Training history emerged as the primary upstream factor, exerting the strongest influence on motor performance (*β* = 0.53), suggesting a cascading developmental effect from sustained physical training to higher-order psychosocial outcomes.

**Table 4:** Structural equation modeling (SEM) – direct effects with latent variables.

Pathway	Estimate	SE	*p*-value	95% CI
Motor Performance → Cognitive Function	0.49	0.06	<0.001***	[0.37, 0.61]
Cognitive Function → Psychological Resilience	0.31	0.07	0.002**	[0.17, 0.45]
Social Engagement → Psychological Resilience	0.28	0.08	0.004**	[0.12, 0.44]
Motor Performance → Social Engagement	0.37	0.05	<0.001***	[0.27, 0.47]
Training History → Motor Performance	0.53	0.05	<0.001***	[0.43, 0.63]

**p < 0.01. ***p < 0.001.

Indirect pathways further supported the developmental cascade linking training to psychosocial outcomes (Table 5). Motor performance influenced psychological resilience through its positive effect on social engagement (*β* = 0.24, 95% CI [0.10, 0.38]), while cognitive function contributed to resilience indirectly by reducing perceived stress (*β* = 0.20, 95% CI [0.07, 0.34]). Training history also exerted an indirect effect on cognitive outcomes via motor performance (*β* = 0.29, 95% CI [0.15, 0.42]), underscoring its foundational role in shaping both physical and cognitive development.

**Table 5:** Structural equation modeling (SEM) – indirect effects with bootstrapped CI.

Indirect pathway	Estimate	Bootstrapped 95% CI	*p*-value
Motor Performance → Social Engagement → Psychological Resilience	0.24	[0.10, 0.38]	0.005**
Cognitive Function → Psychological Resilience → Stress Reduction	0.20	[0.07, 0.34]	0.009**
Training History → Motor → Cognitive Function	0.29	[0.15, 0.42]	0.002**

**p < 0.01.

Moderation analysis revealed contextual factors that influenced the strength of key relationships (Table 6). Higher stress levels weakened the link between motor performance and psychological resilience (*β* = −0.22), suggesting that physiological gains translate less effectively into emotional coping under pressure. Socioeconomic status amplified the effect of social engagement on psychological growth (*β* = 0.26), with stronger benefits observed in higher-SES children. Cognitive load reduced the impact of training history on cognitive performance (*β* = −0.23), indicating that heavy mental demands may blunt the cognitive returns of physical training.

**Table 6:** Moderation effects.

Moderator	Interaction term	Estimate	SE	Sig.	95% CI
Stress Levels	Motor Performance → Psychological Resilience	−0.22	0.06	0.008**	[−0.34, −0.10]
Socioeconomic Status	Social Engagement → Psychological Growth	0.26	0.08	0.004**	[0.10, 0.42]
Cognitive Load	Training History → Cognitive Performance	−0.23	0.07	0.007**	[−0.35, −0.11]

**p < .01.

**Figure 7:**
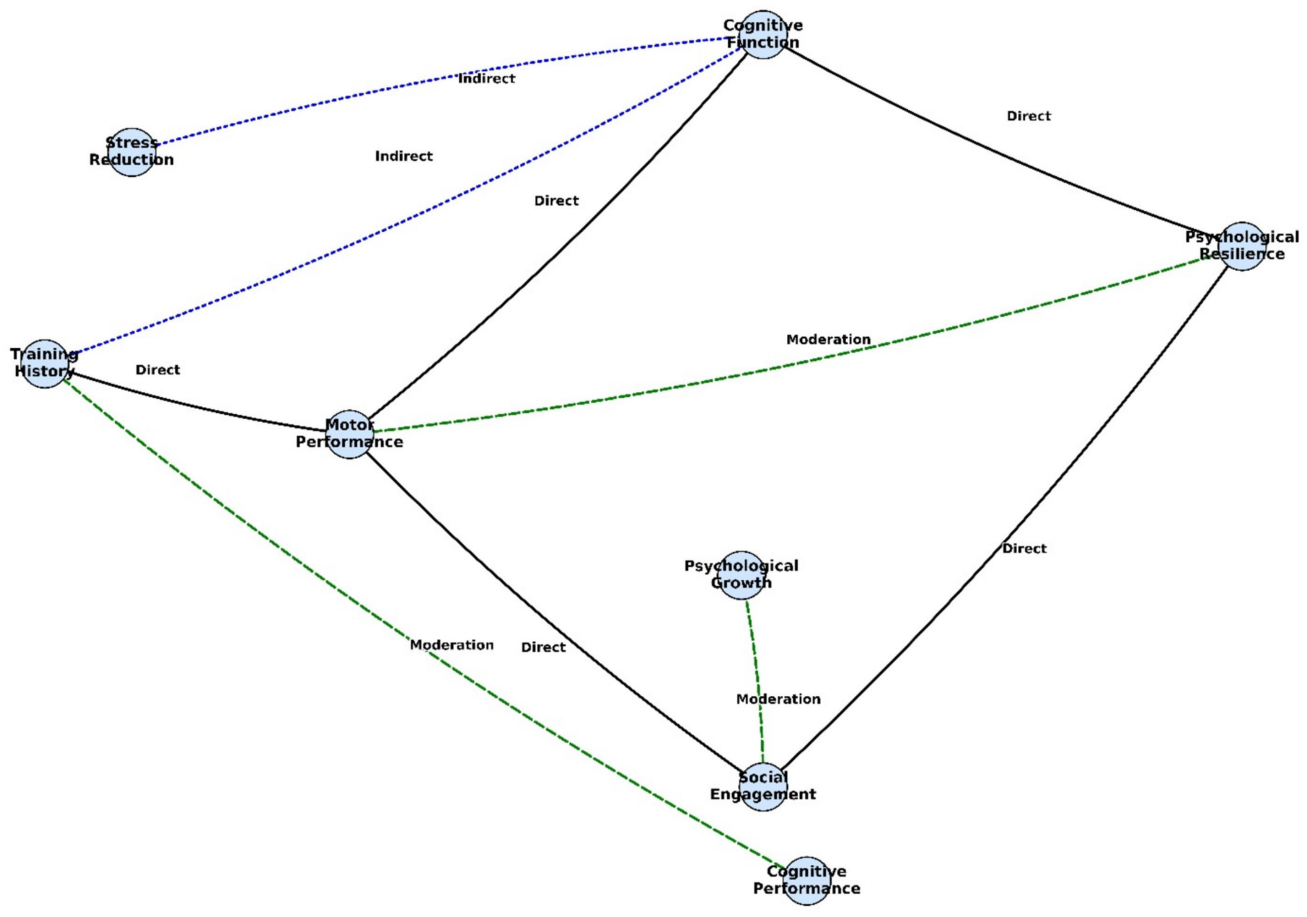
SEM path diagrams for direct (black solid lines), indirect (blue dotted lines), and moderating effects (green dotted lines).

The structural equation model demonstrated excellent overall fit (χ^2^/df = 2.35, CFI = 0.95, RMSEA = 0.04), confirming the robustness of the hypothesized pathways (Figure 7; Table 7). Direct effects supported strong links from motor skill performance to both cognitive and psychosocial outcomes. Mediation paths showed that cognitive function partially explained the effect of motor skills on self-efficacy, while moderation analysis revealed that social engagement buffered the relationship between cognitive function and perceived stress. The model captures the integrated influence of physical, cognitive, and social processes in table tennis-trained children.

**Table 7:** Structural equation modeling (SEM) – model fit indices.

Fit index	Value	Acceptable threshold	Interpretation
χ^2^/df	2.35	<3.0	Good Fit
CFI	0.95	>0.90	Excellent Fit
TLI	0.94	>0.90	Good Fit
RMSEA	0.04	<0.06	Excellent Fit
SRMR	0.05	<0.08	Good Fit

Children with longer training history demonstrated more socially engaged behaviors during play. Higher levels of eye contact, verbal turn-taking, and cooperative interaction were all positively associated with training duration (*r* values = 0.39–0.45), suggesting enhanced communication and group coordination. Increased non-verbal gestures and more frequent offers of assistance (*r* ≈ 0.38–0.41) further reflected prosocial responsiveness. Proximity to peers was negatively correlated (*r* = −0.36), indicating that more experienced players maintained closer physical alignment during cooperative tasks potentially reflecting greater social attunement and spatial awareness.

## 4 Discussion

This study found that table tennis participation was consistently associated with enhanced motor coordination, executive functioning, and psychosocial adaptation in school-aged children. Children with longer training exposure demonstrated more efficient reaction times, improved postural control, and greater visual–motor integration. In the cognitive domain, fewer perseverative errors and faster Stroop responses suggest greater flexibility and attentional control, which may reflect the complex, fast-paced, and anticipatory demands of table tennis play. Socially, higher levels of self-efficacy and cooperation, alongside reduced antisocial behaviors, indicate that sustained participation may foster adaptive interpersonal behaviors and emotional regulation. These findings align with prior studies reporting psychosocial and cognitive benefits in open-skill sports and support the relevance of table tennis as a developmental platform.

The observed gains in motor performance parallel prior findings by Gu et al. (24), who reported improvements in balance and object control; our data (e.g., enhanced agility) align with these outcomes. Consistent with this pattern, Ochs et al. (33) found that table tennis athletes exhibit superior reaction speed and footwork precision, underscoring the sport’s contribution to dynamic motor efficiency through complex, repetitive movement patterns. Mechanistically, such improvements may be driven by high-speed bilateral footwork, anticipatory motor planning, and proprioceptive feedback loops inherent to play. During rallies, athletes continuously adapt to unpredictable ball trajectories, necessitating rapid weight shifts, directional changes, and fine-motor corrections—processes associated with increased cerebellar engagement and enhanced sensorimotor integration (31, 54). Prior findings support our psychosocial results. Martin-Rodriguez et al. (5) reported lower cortisol and higher self-efficacy in table tennis players, while Khudair et al. (32) emphasized improved social regulation. In our sample, longer training duration was associated with greater self-efficacy, better cooperation, and reduced antisocial behaviors. These trends suggest the sport may promote psychosocial development, particularly emotional regulation and prosocial interaction.

Our findings contribute to the literature highlighting the school-based benefits of table tennis. Consistent with prior work showing improved physical education engagement and focus (6) and enhanced postural control (24), we observed parallel associations, including faster reaction times and greater postural stability, indicating that table tennis may serve as a practical intervention for bolstering children’s physical and attentional outcomes. The observed gains in postural control further position the sport as a potential support for children with motor or visual challenges. Stability improvements reported with targeted table tennis drills (31) align with our reductions in center-of-pressure displacement. Moreover, visual–motor enhancements—such as improved fixation (*r* = 0.39) and reduced saccadic latency—mirror findings by Liu et al. (11), suggesting benefits for neuromuscular and oculomotor development.

Our findings converge with prior work indicating that table tennis participation supports physical health, motor performance, and visual function while posing minimal musculoskeletal risk (23, 40, 41). Pradas et al. (4) reported that table tennis players exhibited 5.3% higher bone mineral content and lower fat mass than non-players, underscoring the sport’s contribution to healthy body composition. Similarly, in our sample, longer training duration correlated positively with agility and grip strength, suggesting benefits for endurance and coordination rather than muscular hypertrophy. Complementing these results, Liu et al. (11) documented gains in visual acuity and slower myopia progression among players; we observed parallel improvements, including greater fixation stability and reduced saccadic latency. Notably, Driban et al. (35) found no elevated risk of thumb osteoarthritis in table tennis athletes, aligning with our evidence that sustained participation does not compromise joint health, thereby supporting the sport’s safety and long-term sustainability.

The SEM results indicated that motor performance predicted both cognitive function and social engagement, which in turn predicted psychological resilience. These findings align with Cavalcante Neto et al. (34), who reported gains in motor planning and cognitive processing following table tennis training, and with Luca et al. (25), who observed improved cardiovascular efficiency and reduced anxiety, highlighting the sport’s multidimensional benefits. Moderation analyses further clarified boundary conditions: higher stress attenuated the association between motor skills and resilience, whereas higher socioeconomic status strengthened the link between social engagement and psychological growth. Consistent with Khudair et al. (32), academic stressors diminished training-related cognitive gains. Collectively, the pattern suggests that improved motor performance may augment cognitive function and social interaction by engaging neural circuits supporting executive control, coordination, and social cognition; these enhancements, in turn, appear to cultivate psychological resilience through strengthened self-regulation and adaptive coping under training-induced stress.

The moderation results underscore the salience of contextual supports. Elevated stress attenuated training-related gains, suggesting that chronic cognitive load competes for attentional resources and impedes consolidation of executive processes (42–47). In contrast, higher socioeconomic status may amplify benefits by enabling structured routines, adequate nutrition, and supportive home environments. These patterns highlight the need for holistic program design that incorporates stress-regulation strategies and promotes equitable access to maximize developmental returns from sport-based interventions (25, 55). A key contribution of this study is the integration of concept mapping with structural equation modeling: concept mapping delineated participant-perceived domains across motor, cognitive, and social dimensions, whereas SEM quantified their interrelations. This dual-method framework offers a novel approach for modeling multidimensional developmental outcomes in sports interventions and is well suited to informing school- and community-based program design.

### 4.1 Limitations and future research

One limitation of this study is that it primarily relied on cross-sectional data, which restricts causal interpretations of the relationships observed. Although the sample was large, well-balanced by sex, and covered a broad age range, all participants were recruited from a single cultural and educational context in urban China. This may limit the generalizability of findings to children from other cultural, rural, or socioeconomic contexts, particularly in terms of psychosocial norms, physical education practices, and training access. Future studies should replicate the design in diverse national settings to evaluate cross-cultural applicability, especially regarding concept mapping feasibility and psychosocial interpretations of table tennis participation. In future research, we will conduct a longitudinal study to assess how table tennis participation influences cognitive, motor, and psychological development over time.

Although all cognitive and psychosocial instruments used in this study have been previously validated in Chinese or Mandarin-speaking populations including the WCST, Stroop Color-Word Test, PSQ-20, and a shortened version of the SSBS-2 some were originally developed in Western contexts. Despite strong psychometric evidence in Chinese cohorts, potential residual cultural variance in construct interpretation or response behavior cannot be ruled out. Therefore, while measurement validity is supported, future studies may consider additional cross-cultural confirmatory factor analyses to strengthen generalizability. Additionally, we will incorporate neurophysiological assessments, such as functional MRI and electroencephalography, to provide deeper insights into the neural mechanisms underlying the cognitive and motor benefits of table tennis training.

## 5 Conclusion

This study suggests that table tennis may serve as a scalable, low-cost intervention to support motor, cognitive, and psychosocial development in children. Associations with agility, attentional control, and social engagement reflect the sport’s integrative demands. While the cross-sectional design limits causal interpretation, the pattern of results highlights the potential of table tennis to complement existing school-based developmental programs, particularly in settings where time, equipment, and space are limited. Longer training durations were associated with faster reaction times, improved postural control, enhanced Stroop and WCST performance, and superior anticipatory gaze. Social–emotional indicators such as self-efficacy, cooperation, and stress regulation also improved. Taken together, these findings position table tennis as a promising developmental platform that integrates neuromuscular coordination, cognitive flexibility, and peer engagement. Its scalability and adaptability make it a viable candidate for integration into urban educational settings, particularly where time and physical space are constrained.

## Data Availability

The original contributions presented in the study are included in the article/Supplementary material, further inquiries can be directed to the corresponding author.
