# Supplementary material for: Multidisciplinary correlates of table tennis participation in children: a concept mapping study
Source: Front Public Health. 2025 Oct 1;13:1644306. doi: 10.3389/fpubh.2025.1644306 (PMC12521237; doi:10.3389/fpubh.2025.1644306)
Supplement: Supplementary file 1 [file Data_Sheet_1.docx]

**Table S1. Subgroup Comparisons by Sex and Age Group for Key Training-Associated Outcomes**

| **Domain** | **Variable** | **Males (n=156)** | **Females (n=156)** | **p (Sex)** | **Age 8–10 (n=141)** | **Age 11–14 (n=171)** | **p (Age Group)** |
| --- | --- | --- | --- | --- | --- | --- | --- |
| **Motor Skills** | Agility (s) | 12.0 ± 1.6 | 12.7 ± 1.7 | 0.049 | 11.8 ± 1.3 | 12.8 ± 1.5 | <0.001 |
|  | Balance CoP (mm) | 13.4 ± 2.7 | 13.1 ± 2.8 | 0.421 | 12.6 ± 2.4 | 13.8 ± 2.9 | 0.018 |
| **Cognitive** | WCST Completion Time (s) | 59.2 ± 11.8 | 57.6 ± 12.3 | 0.312 | 61.0 ± 13.4 | 55.3 ± 10.8 | 0.004 |
|  | Stroop Interference (ms) | 203.4 ± 49.1 | 180.2 ± 42.7 | 0.042 | 205.7 ± 45.8 | 176.3 ± 43.1 | 0.008 |
| **Psychosocial** | Cooperation (SSBS‑2) | 3.81 ± 0.47 | 4.02 ± 0.41 | 0.038 | 3.74 ± 0.39 | 4.03 ± 0.48 | 0.016 |
|  | Antisocial Behavior (SSBS‑2) | 1.91 ± 0.29 | 1.87 ± 0.33 | 0.572 | 1.96 ± 0.27 | 1.83 ± 0.31 | 0.037 |

**Note**: Values are reported as mean ± SD. *p*-values reflect independent t-tests between sex or age groups. All comparisons are exploratory and not corrected for multiplicity. No significant sex × age or sex × training duration interactions were observed.

**Table S2:** Postural Stability Performance and Correlations with Training Duration

| **Condition** | **CoP Displacement (mm) ± SD** | **r** | **p-value** | **95% CI** |
| --- | --- | --- | --- | --- |
| Eyes Open, Feet Together | 4.2 ± 1.1 | -0.39 | <0.001*** | [-0.52, -0.25] |
| Eyes Closed, Feet Together | 6.7 ± 1.5 | -0.42 | <0.001*** | [-0.55, -0.29] |
| Single-Leg Stance (Dominant Leg) | 10.5 ± 2.3 | -0.44 | <0.001*** | [-0.57, -0.31] |
| Single-Leg Stance (Non-Dominant Leg) | 11.2 ± 2.6 | -0.46 | <0.001*** | [-0.59, -0.33] |
| Mean Mediolateral CoP Displacement | 7.8 ± 1.9 | -0.40 | <0.001*** | [-0.53, -0.26] |
| Mean Anteroposterior CoP Displacement | 8.3 ± 2.1 | -0.43 | <0.001*** | [-0.56, -0.30] |

**Note:** *p < 0.05, **p < 0.01, ***p < 0.001.*


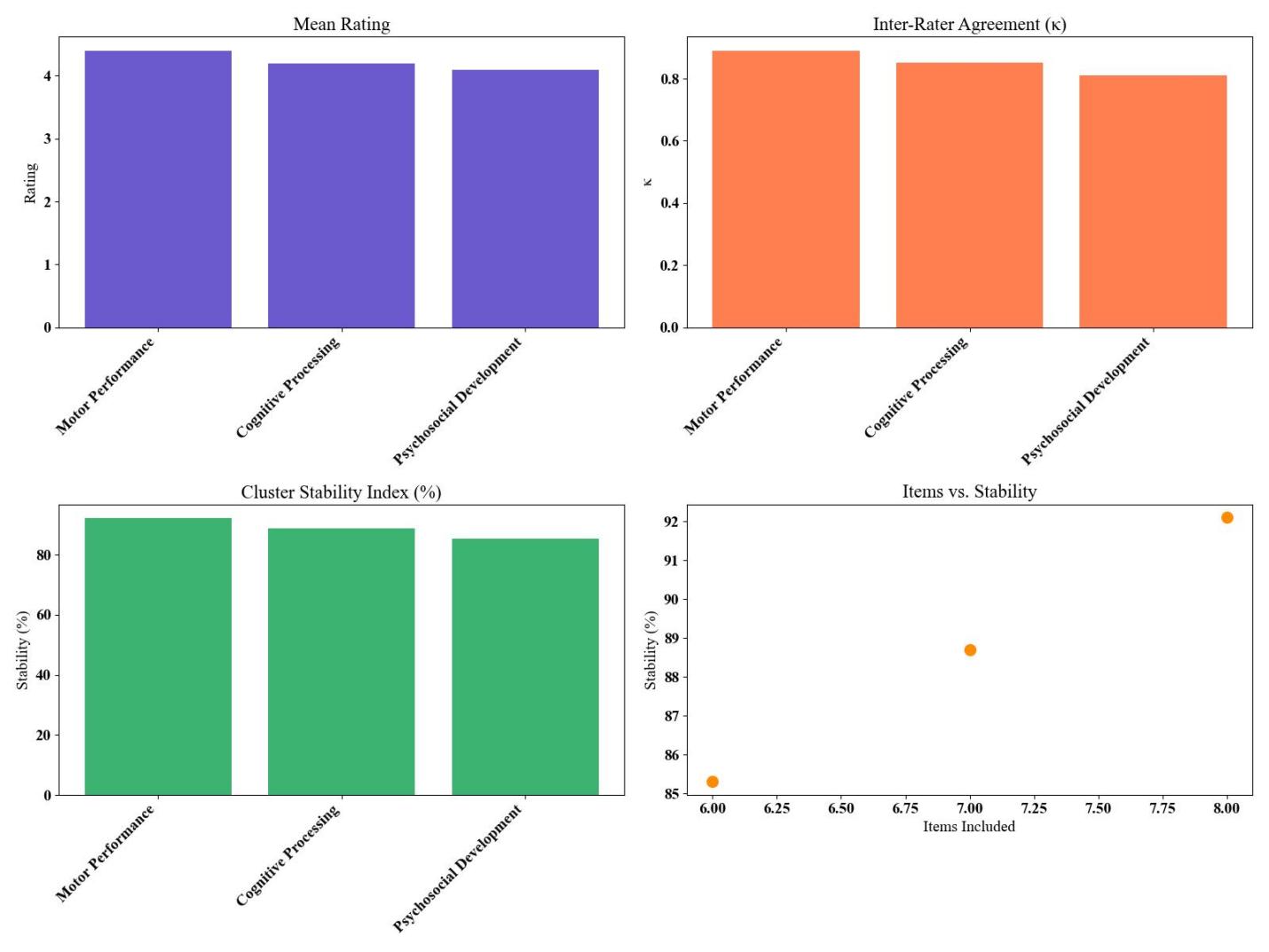


**Figure S1:** Rating and Sorting Analysis Using Hierarchical Clustering Agreement (HCA) showing a) bar chart of mean ratings per cluster, b) bar chart of inter-rater agreement (κ), c) bar chart of cluster stability index (%), and d) scatter plot illustrating the relationship between number of items and cluster stability.


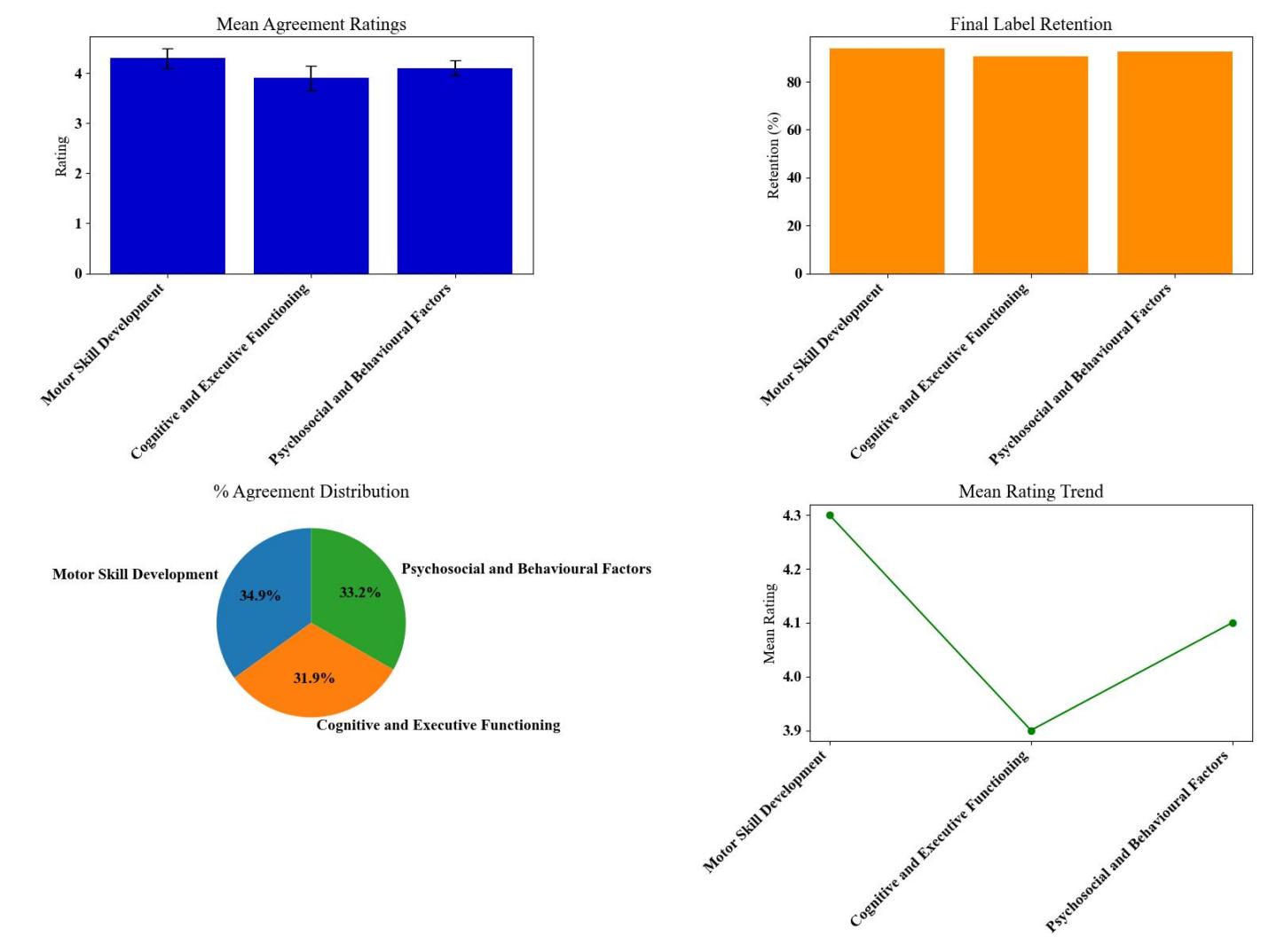


**Figure S2:** Label Agreement Scores showing a) bar chart of mean agreement ratings with standard deviation per cluster, b) bar chart of final label retention percentages, c) pie chart of agreement distribution across clusters, and d) line plot of mean ratings across clusters.


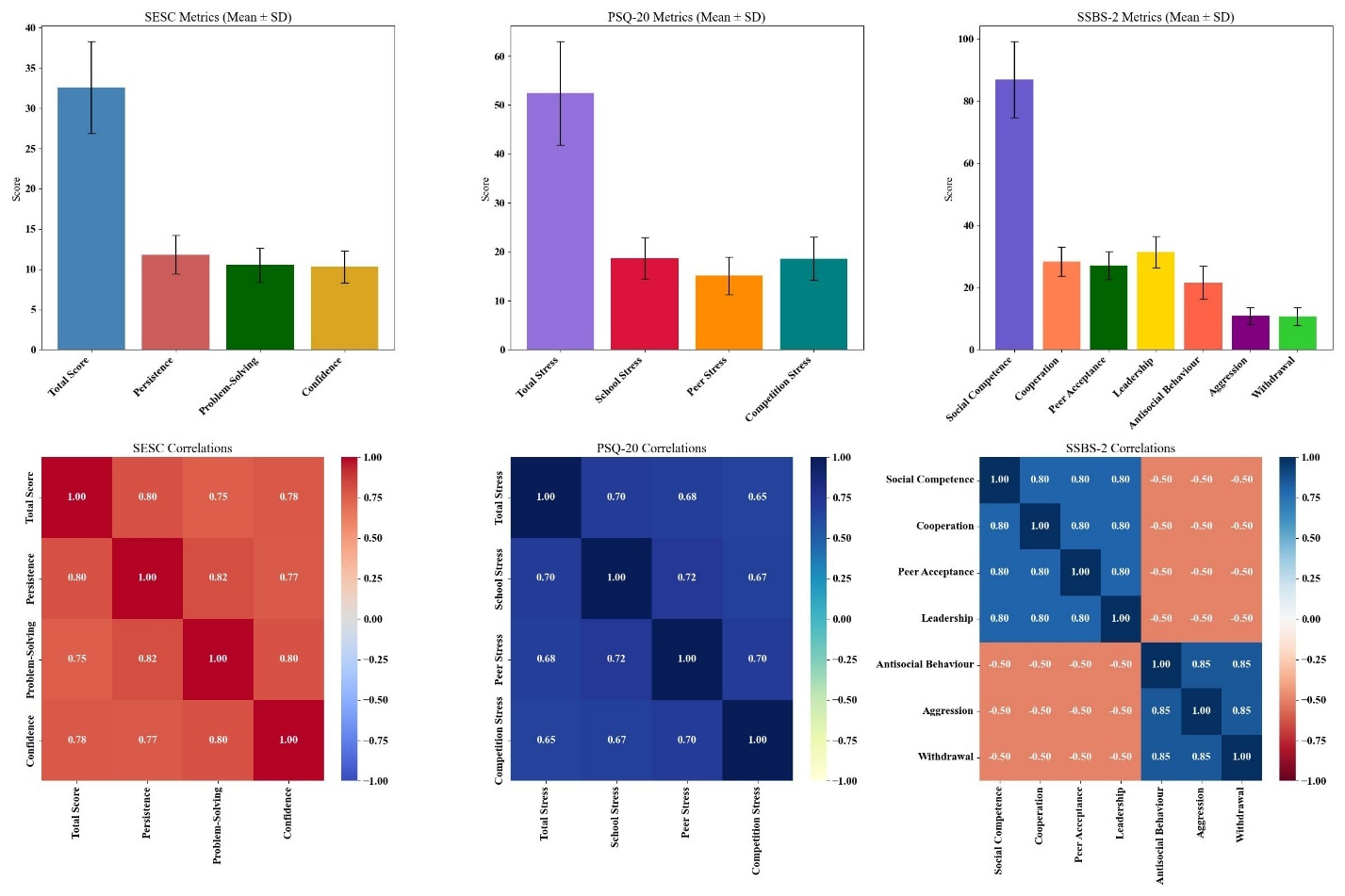


**Figure S3:** Psychological Stress, Self-Efficacy, and Social Engagement Measures and Correlations a) SESC metrics. b) PSQ-20 metrics. c) SSBS-2 metrics. d) SESC correlation matrix. e) PSQ-20 correlation matrix. f) SSBS-2 correlation matrix.


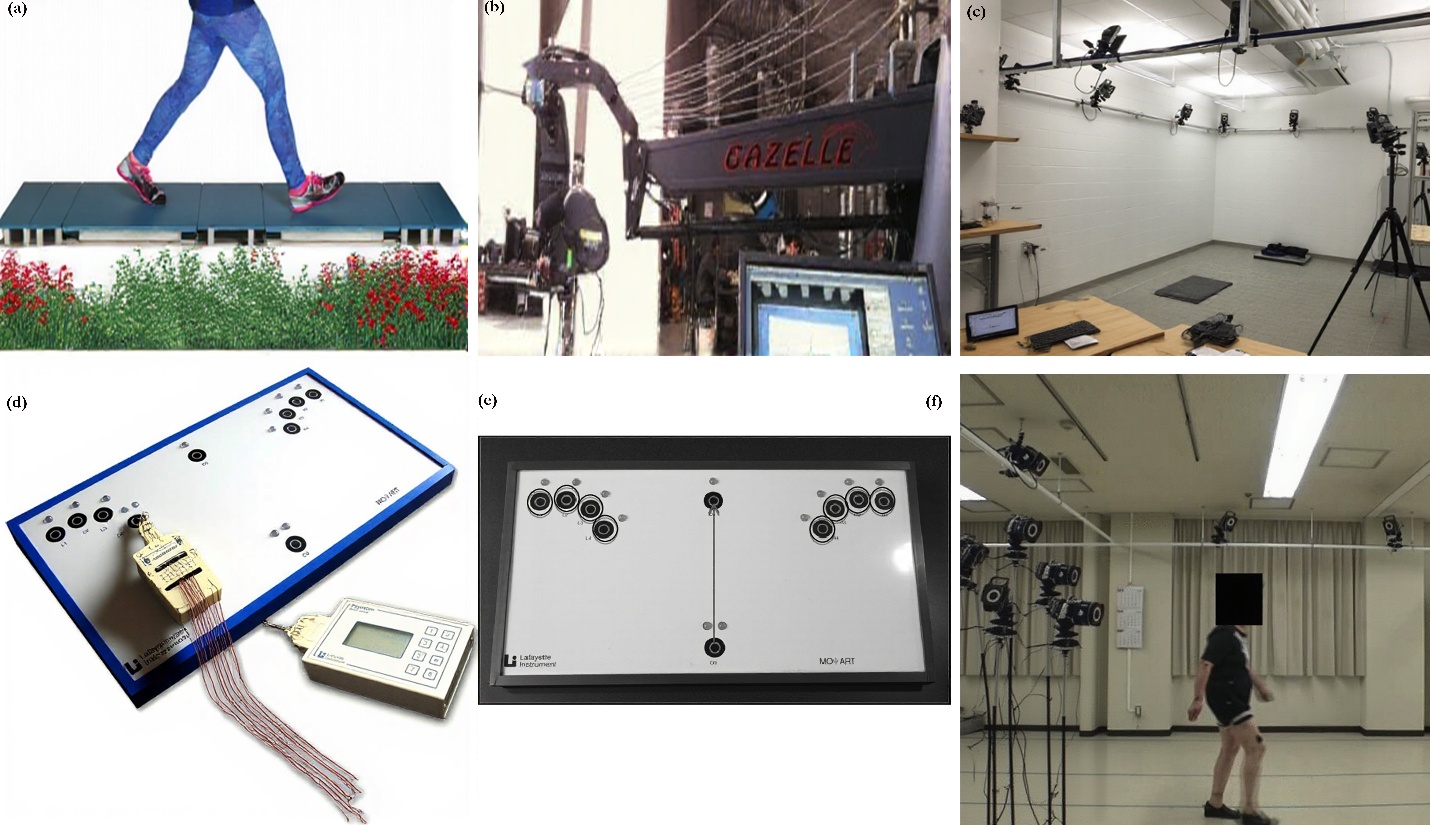


**Figure S4:** Experimental setup showing (a) force platform for balance, (b) Gazelle Sports system for hand-eye coordination, (c) motion capture lab, (d) reaction-time device, (e) cognitive-motor integration board, and (f) participant under 3D motion capture.


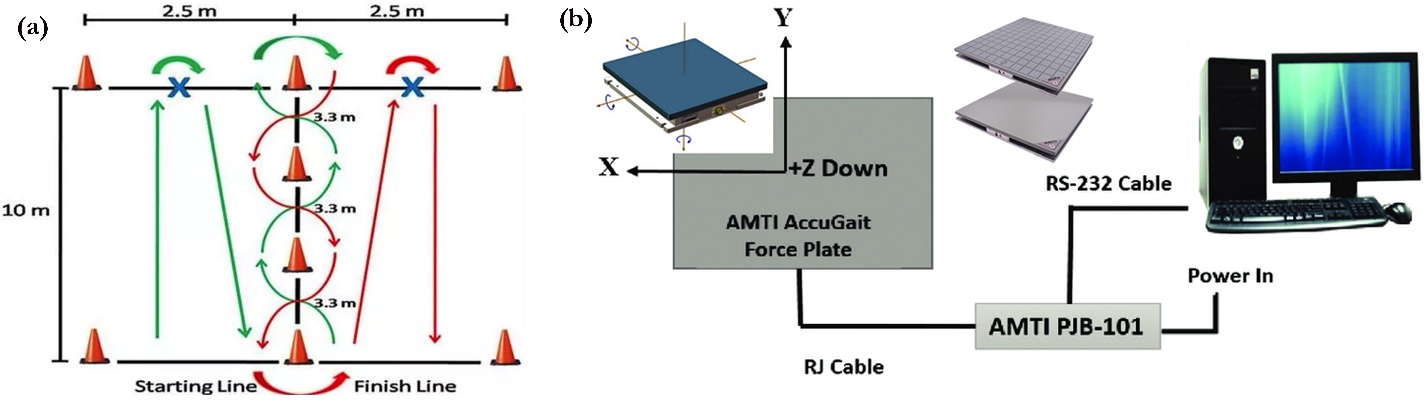


**Figure S5:** Experimental setup for motor and balance assessments, including (a) Illinois Agility Test course layout with directional movement patterns and (b) AMTI AccuGait force plate system with data acquisition components for postural stability analysis.
